# Hollow-degree-tuned Prussian blue–mesoporous silica nanoplatforms for chemo-photothermal cancer therapy with preliminary microfluidic perfusion evaluation

**DOI:** 10.3389/fbioe.2026.1932857

**Published:** 2026-09-14

**Authors:** Jingjing Xi, Jingjing Liu, Heyao Zhang, Mengxi Wang, Chenyi Lu, Tinghan Wang, Xijun Piao, Ri Huang, Keyun Ren, Xiao Mei

**Affiliations:** 1 Center of Self-Propelled Nanotechnologies, Suzhou Industrial Park Institute of Services Outsourcing, Suzhou, China; 2 Suzhou Huitong Chromatographic Separation and Purification Co., Ltd., Suzhou, China; 3 Catug Biotechnology (Suzhou) Co., Ltd., Suzhou, China

**Keywords:** chemo-photothermal therapy, drug delivery, hollow-degree engineering, mesoporous silica, microfluidic evaluation, prussian blue

## Abstract

**Background:**

Balancing photothermal performance and drug-delivery capacity remains a key challenge in the structural design of multifunctional nanomedicines. Hollowing can increase internal space for drug accommodation but may simultaneously reduce the amount of NIR-absorbing Prussian blue (PB), creating an inherent trade-off between drug-delivery capacity and photothermal performance.

**Methods:**

The hollow state of hollow mesoporous Prussian blue (HMPB) nanocubes was regulated by controlled HCl etching, followed by a common mesoporous silica (mSiO_2_) coating, to generate low-, intermediate-, and high-hollow formulations. The resulting formulations were evaluated for physicochemical properties, photothermal performance, doxorubicin (DOX) loading and release, cellular response, intracellular fluorescence, chemo-photothermal interaction, and preliminary microfluidic perfusion performance.

**Results:**

Increasing hollowing enhanced DOX loading from 525 to 867 μg⋅mg^−1^ and promoted drug release, but progressively reduced photothermal performance. The measured photothermal conversion efficiencies of 1-, 2-, and 3-HMPB@mSiO_2_ were 23.37%, 18.62%, and 14.41%, respectively. ICP-MS showed decreasing Fe contents with increasing hollowing, while PB-equivalent-mass-matched measurements yielded similar photothermal conversion efficiencies (16.80%, 15.80%, and 14.41%), indicating that PB abundance is a major contributor to the observed photothermal differences. DOX release was enhanced by both mild acidity and mild hyperthermia relative to pH 7.4/37 °C. After treatment-matched correction for nanoparticle-related optical interference, the blank carriers showed limited acute carrier-associated cytotoxicity in HeLa cells over 24 h, and quantitative fluorescence imaging confirmed effective intracellular DOX delivery. At an equal total formulation concentration, the intermediate-hollow 2-HMPB@mSiO_2_-DOX formulation produced the strongest combined therapeutic response. Multi-dose combination-index, Bliss-independence, and Loewe-additivity analyses further supported increasingly synergistic chemo-photothermal interactions with increasing treatment effect. Preliminary syringe-pump-driven microfluidic perfusion in physiological medium preserved the principal structure-dependent trends in particle dispersion, drug leakage, and NIR heating.

**Conclusion:**

These results demonstrate that hollow-state engineering provides a practical strategy for balancing competing photothermal and drug-delivery functions in PB-based nanoplatforms.

## 1 Introduction

Current clinical cancer treatments include surgery, chemotherapy, radiotherapy, targeted therapy, and immunotherapy (Joosten et al., 2021; D’Alterio et al., 2020; Zheng et al., 2023; Hawryłkiewicz and Ptaszyńska, 2021; Zhou et al., 2023). Despite major advances, monotherapies remain limited by incomplete responses, treatment-related toxicity, recurrence, and intrinsic or acquired drug resistance (Chen et al., 2022; Magdy et al., 2020; Yang et al., 2023; Pratikshya et al., 2023; Pi et al., 2022). Drug resistance can arise from inefficient drug delivery, tumor heterogeneity, altered gene expression, and an immunosuppressive tumor microenvironment (Zheng et al., 2024; Uckun and Qazi, 2022; Tiek and Cheng, 2022).

Several strategies have been developed to address these limitations, including nanocarrier-mediated drug delivery (Sun et al., 2023; Yi et al., 2023), personalized therapy (Balakrishnan et al., 2023; Gao et al., 2023), tumor-microenvironment modulation (Mondal et al., 2022; Schwartz et al., 2017), and multimodal treatment (Pei et al., 2023; Jang et al., 2015; Lin et al., 2019). Nanomaterial-based platforms are particularly attractive because they can integrate chemotherapy with photothermal, photodynamic, immunological, or radiotherapeutic functions (Dong et al., 2013; Zhou et al., 2020; Zeng et al., 2020; Chang et al., 2017). Microfluidic and organ-on-chip systems further provide controlled-flow and microphysiological environments for evaluating nanocarrier transport and therapeutic response beyond conventional static culture (Sun et al., 2024; Zhai et al., 2024; Huh et al., 2012; Bhatia and Ingber, 2014; Sackmann et al., 2014).

Prussian blue (PB) is an established near-infrared (NIR)-responsive material for photothermal therapy, imaging, and drug delivery (Wang et al., 2022; Zakaria et al., 2015; Wang et al., 2013; Shi et al., 2024; Guo et al., 2024; Zhang et al., 2025; Fu et al., 2012). Its optical response, colloidal behavior, and biological functionality can be tuned through metal-ion doping, polymer modification, and structural engineering (Cahu et al., 2021; Shou et al., 2020; Azhar et al., 2018; Cheng et al., 2014). Hollow PB nanocages have also served as drug reservoirs for combined chemotherapy and photothermal therapy (Zakaria et al., 2015; Wang et al., 2013; Shi et al., 2024; Guo et al., 2024; Zhang et al., 2025; Wu et al., 2015; Bai et al., 2021). In parallel, mesoporous silica has been integrated with PB to provide porous drug-loading interfaces, surface stabilization, and additional functionalization options (Giacomo et al., 2017; Qian and Yang, 2006; Moorthy et al., 2018; Sahoo et al., 2022).

Most PB-based therapeutic nanoplatforms are optimized around a fixed particle architecture, surface modification, or payload. Hollowing, however, introduces an inherent design trade-off. Removing PB can reduce the amount of NIR-absorbing material, whereas the newly created internal space can increase drug accommodation. Photothermal output and drug-delivery capacity may therefore change in opposite directions as hollowing progresses. Our previous HMPB@mSiO_2_ platform demonstrated the feasibility of combining a hollow mesoporous PB core with an mSiO_2_ shell for drug delivery and photothermal treatment (Mei et al., 2022). However, hollow-state-dependent structure-property relationships were not systematically compared.

We therefore hypothesized that lower hollowing would favor PB-dependent photothermal performance, whereas greater hollowing would favor drug loading and release. If these opposing trends coexist, an intermediate hollow architecture should provide a more favorable functional balance for chemo-photothermal combination therapy than either structural extreme (Figure 1).

**FIGURE 1:**
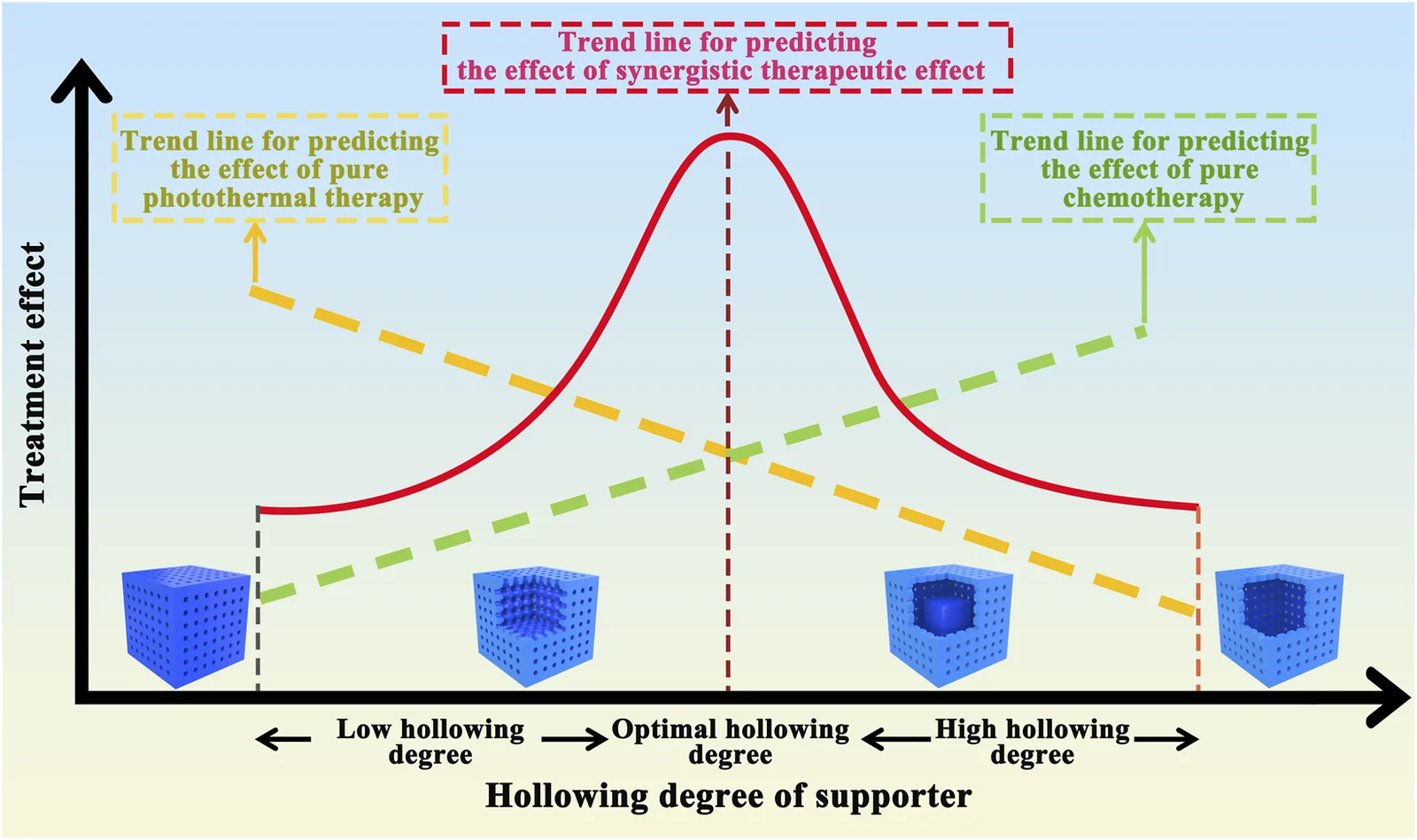
Conceptual illustration of the working hypothesis for balancing chemotherapy and photothermal therapy through hollow-degree engineering.

To test this hypothesis, HCl-mediated etching was used to generate three benchmark HMPB hollow states, each followed by the same mSiO_2_-coating procedure. We compared morphology, physicochemical properties, photothermal conversion, DOX loading and release, cellular response, intracellular fluorescence, and chemo-photothermal interaction. A broader HCl-etching screen and independent repeat syntheses were included to define the accessible morphology range and assess structural reproducibility. Preliminary microfluidic perfusion was then used to examine whether the principal material trends were retained under flow.

## 2 Materials and methods

### 2.1 Materials

Potassium ferricyanide (K_3_[Fe(CN)_6_]) was purchased from Shanghai Aladdin Biochemical Technology Co., Ltd. Polyvinylpyrrolidone (PVP, K30), cetyltrimethylammonium bromide (CTAB, ≥99%), and doxorubicin (DOX) were obtained from Shanghai Macklin Biochemical Co., Ltd. Concentrated ammonia solution (NH_3_·H_2_O, 25-28 wt%), tetraethyl orthosilicate (TEOS, >97 wt%), ethanol (C_2_H_5_OH, >99.7 wt%), dimethyl sulfoxide (DMSO, A.R.), and hydrochloric acid (HCl, 36-37 wt%) were supplied by Sinopharm Chemical Reagent Co., Ltd. Phosphate-buffered saline (PBS) solutions (pH 7.4 and pH 6.0) were acquired from Sankong Biotechnology (Shanghai) Co., Ltd. Cell culture media were provided by Sigma-Aldrich. 3-(4,5-Dimethylthiazol-2-yl)-2,5-diphenyltetrazolium bromide (MTT) was sourced from Keygen Biotech. Hoechst 33342 nuclear stain was procured from Thermo Scientific. All chemicals were used as received without further purification.

### 2.2 Synthesis of n-HMPB nanocubes with different hollow states

K_3_[Fe(CN)_6_] (528 mg) and PVP (12 g) were dissolved in 160 mL of 0.01 M HCl with stirring at 25 °C for 30 min until a transparent solution formed. The mixture was transferred to a Teflon-lined stainless-steel autoclave and heated at 80 °C for 24 h. After cooling to room temperature, the precipitate was collected by centrifugation (12,000 rpm, 15 min), washed three times each with deionized water and ethanol, and vacuum-dried at 40 °C for 24 h to obtain mesoporous Prussian blue (MPB) nanocubes (Mei et al., 2022).

The obtained MPB (240 mg) was divided into three 80 mg aliquots. Each aliquot was mixed with PVP (200 mg) and dispersed in 40 mL of HCl at 0.5, 0.75, or 1.0 M. After 30 min of ultrasonication and 2 h of magnetic stirring, the suspensions were transferred to Teflon-lined autoclaves and heated at 140 °C for 4 h. After cooling, the products were collected by centrifugation (12,000 rpm, 20 min), washed three times each with deionized water and ethanol, and vacuum-dried overnight. The products were designated 1-HMPB, 2-HMPB, and 3-HMPB, respectively.

### 2.3 Synthesis of n-HMPB@mSiO_2_ nanocubes with different hollow states

Each HMPB sample (5 mg) was dispersed in a mixture of CTAB (1 g) and absolute ethanol (45 mL) and sonicated for 30 min. Deionized water (560 mL) was added, and the dispersion was stirred at 35 °C for 1 h. TEOS (625 µL) and NH_3_·H_2_O (62.5 µL) were then added dropwise, and the reaction was continued at 48 °C for 48 h with stirring. The solids were collected by centrifugation (10,000 rpm, 10 min), washed three times each with deionized water and ethanol, and subjected to three 6 h reflux cycles in 500 mL ethanol/HCl (1:500, v/v) at 60 °C to extract CTAB. The final products were collected by centrifugation, vacuum-dried, and designated 1-HMPB@mSiO_2_, 2-HMPB@mSiO_2_, and 3-HMPB@mSiO_2_ (Mei et al., 2022).

### 2.4 *In Vitro* DOX loading and release experiments

DOX was loaded by free diffusion. Each n-HMPB@mSiO_2_ sample (5 mg) was mixed with DOX (5 mg) in 5 mL PBS (pH 7.4) and shaken at 100 rpm at room temperature for 24 h. After centrifugation, the supernatant was collected, and the precipitate was washed three times with 5 mL PBS. The supernatant and washings were pooled and adjusted to 25 mL. A 2.5 mL aliquot was analyzed by UV-vis spectroscopy. Unloaded DOX was quantified using a calibration curve prepared with 0, 6.25, 12.5, 25, 50, and 100 μg·mL^-1^ DOX. The total unloaded amount was calculated from the measured concentration and the 25 mL volume, and loaded DOX was obtained by difference from the initial 5 mg. Drug-loading content (DLC) and encapsulation efficiency (EE) were calculated relative to the initial carrier mass and initial DOX amount, respectively. To assess batch-to-batch reproducibility, two additional batches of each formulation were independently synthesized and subjected to the same loading procedure.

For release testing, n-HMPB@mSiO_2_-DOX was evaluated under four conditions: PBS at pH 7.4/37 °C, pH 7.4/42.5 °C, pH 6.0/37 °C, and pH 6.0/42.5 °C. Drug-loaded particles were dispersed in 20 mL PBS for each condition. At 0, 0.5, 1, 2, 4, 8, 12, 24, and 48 h, the samples were centrifuged and a 2.0 mL aliquot of clear supernatant was analyzed by UV-vis spectroscopy at 482 nm. The aliquot was then returned to the release system, and the nanoparticles were redispersed for continued incubation. Released DOX was calculated from the measured concentration and the 2.0 mL analytical volume (Mei et al., 2022).

### 2.5 Photothermal performance measurements

Photothermal heating was evaluated with n-HMPB@mSiO_2_ dispersions in deionized water. Concentration-dependent heating was measured at 0, 100, 200, 500, and 1,000 μg·mL^-1^ under 808 nm irradiation (1 W·cm^-2^, 5 min). Power-dependent heating was measured with 500 μg·mL^-1^ dispersions at 0.5, 1.0, and 2.0 W·cm^-2^ for 5 min. Photothermal cycling was evaluated over three ON/OFF cycles using 500 μg·mL^-1^ dispersions; each cycle consisted of 5 min irradiation at 2.0 W·cm^-2^ followed by 10 min of natural cooling. Temperature was recorded with an infrared thermal camera. For photothermal conversion-efficiency measurements, 200 μL of each dispersion (200 μg·mL^-1^) was placed in a 96-well plate and irradiated vertically at an actual incident power of 1.0 W (2.5 W·cm^-2^) for 600 s, followed by 600 s of natural cooling. The absorbance at 808 nm (A_808_) was measured for the same dispersions, and deionized water served as the blank. Photothermal conversion efficiency (η) was calculated from the heating and cooling data using a method adapted from Hoffman et al. (2014). The same predefined 60–480 s cooling interval was used for linear regression for all formulations. Detailed equations are provided in the Supplementary Material.

To experimentally assess the contribution of PB abundance to photothermal performance, an additional PB-equivalent-mass-matched measurement was performed. The PB-equivalent mass fractions of 1-, 2-, and 3-HMPB@mSiO_2_ were estimated from the ICP-MS-measured Fe contents using the theoretical Fe mass fraction of anhydrous Prussian blue, Fe_4_[Fe(CN)_6_]_3_ (0.4550). Based on the measured Fe contents, the calculated PB-equivalent mass fractions were 69.01%, 58.46%, and 46.81%, respectively. The PB-equivalent mass contained in 200 μL of a 200 μg·mL^-1^ 3-HMPB@mSiO_2_ dispersion was selected as the common reference. Accordingly, 1-, 2-, and 3-HMPB@mSiO_2_ dispersions were prepared at composite concentrations of 135.7, 160.2, and 200.0 μg·mL^−1^, respectively, providing approximately 18.73 μg of PB-equivalent material in each 200 μL sample. Photothermal conversion-efficiency measurements were then repeated under the same irradiation, cooling, absorbance-measurement, and calculation conditions described above.

### 2.6 *In vitro* HeLa cell experiments

#### 2.6.1 Cell culture

HeLa cells were cultured in Dulbecco’s Modified Eagle Medium (DMEM) supplemented with 10% (v/v) fetal bovine serum, 100 U·mL^-1^ penicillin, and 100 µg·mL^-1^ streptomycin. Cells were maintained at 37 °C in a humidified atmosphere containing 5% CO_2_.

#### 2.6.2 *In Vitro* cytotoxicity assay (MTT)

HeLa cells were seeded in 96-well plates at 1 × 10^4^ cells per well in 200 µL culture medium and allowed to attach for 24 h. The medium was then replaced with fresh medium containing 0, 62.5, 125, 250, 500, or 1,000 µg·mL^-1^ n-HMPB@mSiO_2_, and the cells were incubated for another 24 h. The nanoparticle-containing medium was removed, and the wells were washed twice with PBS. Fresh medium containing MTT (20 µL of 5 mg·mL^-1^ MTT per well) was added for 4 h. The supernatant was removed, and 200 µL DMSO was added to dissolve the formazan product. The plate contents were clarified by low-speed centrifugation (250 × g, 5 min), and the supernatant was transferred to a fresh plate for absorbance measurement at 570 nm (Wang et al., 2015; Kroll et al., 2012; Holder et al., 2012). At each nanoparticle concentration, a treatment-matched cells + nanoparticle/no-MTT control underwent the same exposure, washing, DMSO, clarification, and transfer steps and provided the background term that was directly subtracted from the corresponding sample signal. Cell-free nanoparticle + MTT controls were processed in parallel to test direct nanoparticle-MTT reactivity and residual particulate interference after the same analytical workflow. This cell-free control was used as an independent assay-validity check rather than being subtracted a second time from the treatment-matched corrected signal; the correction equations and residual-interference metric are provided in the Supplementary Material. Three independent experiments were performed for each condition. Technical replicates were averaged within each experiment, and data are reported as mean ± SD (n = 3).

#### 2.6.3 *In Vitro* therapeutic cytotoxicity assay (MTT)

HeLa cells were seeded in 96-well plates at 1 × 10^4^ cells per well in 200 µL DMEM and allowed to attach overnight at 37 °C and 5% CO_2_. Treatment groups included blank medium; blank 1-, 2-, or 3-HMPB@mSiO_2_ at 250 µg·mL^-1^; 1-, 2-, or 3-HMPB@mSiO_2_-DOX at 250 µg·mL^-1^; each blank carrier followed by 808 nm irradiation (1 W·cm^-2^, 5 min); and each DOX-loaded carrier followed by the same irradiation.

After treatment, the cells were incubated for 24 h. The treatment medium was removed, the wells were washed twice with PBS, and fresh medium containing MTT (20 µL of 5 mg·mL^-1^ MTT per well) was added for 4 h. The supernatant was then removed, and 200 µL DMSO was added to dissolve the formazan product. The plate contents were clarified by centrifugation (250 × g, 5 min), and the supernatant was transferred to a fresh plate for absorbance measurement at 570 nm. Treatment-matched cells + formulation/no-MTT controls were processed in parallel for each carrier, DOX-loaded carrier, and irradiation condition, and the corresponding no-MTT background was directly subtracted before normalization. Cell-free formulation + MTT controls were processed in parallel as an independent check for direct formulation-MTT reactivity and residual particulate interference. Each treatment group contained six technical replicate wells per experiment, and the experiment was repeated independently three times. Technical replicates were averaged within each experiment, and data are reported as mean ± SD (n = 3).

#### 2.6.4 High-content imaging and mean fluorescence intensity analysis

HeLa cells were seeded in 8-well chamber slides at 1 × 10^4^ cells per well and incubated overnight. Cells were treated with 100 µg·mL^-1^ 2-HMPB@mSiO_2_-DOX, free DOX at the corresponding DOX-equivalent concentration, or DMEM alone. After 4 h at 37 °C, the wells were washed twice with PBS and the nuclei were stained with Hoechst 33342 (5 µg·mL^-1^) for 1 h. DOX and Hoechst fluorescence were acquired on the high-content analysis system using identical exposure settings for all groups (excitation/emission: 485/595 nm for DOX and 350/461 nm for Hoechst 33342). DOX mean fluorescence intensity (MFI) was quantified with the same segmentation and intensity settings across groups and background-corrected to the untreated control. Three independent experiments were performed, and data are reported as mean ± SD (n = 3).

#### 2.6.5 Quantitative analysis of chemo-photothermal synergy

For quantitative interaction analysis, 2-HMPB@mSiO_2_-DOX was tested in a separate multi-dose experiment at total loaded-formulation concentrations of 0, 62.5, 125, 187.5, 250, 312.5, 375, and 500 μg·mL^-1^. Based on the measured drug loading, these concentrations corresponded to DOX-equivalent concentrations of 0, 26.1, 52.1, 78.2, 104.2, 130.3, 156.3, and 208.4 μg·mL^-1^. For PTT, dose was represented by a dimensionless photothermal treatment-intensity factor (x = 0, 0.25, 0.50, 0.75, 1.00, 1.25, 1.50, and 2.00), which simultaneously scaled nanoparticle concentration and NIR fluence at a fixed ratio; 1.00× corresponded to 250 μg·mL^-1^ unloaded 2-HMPB@mSiO_2_ and 300 J·cm^-2^. The matched NIR fluences were 0, 75, 150, 225, 300, 375, 450, and 600 J·cm^-2^, with the nanoparticle concentration scaled proportionally. In the fixed-ratio combination series, chemotherapy and PTT dose axes were scaled by the same x factor. Unloaded 2-HMPB@mSiO_2_ controls were included at the corresponding nanoparticle concentrations. Within each independent experiment, viabilities for chemotherapy, PTT, and the combination were normalized to the dose-matched unloaded-carrier control before averaging. Combination-index (CI) values were calculated in this fixed-ratio dose-factor space using the Chou-Talalay median-effect framework; CI < 1, CI = 1, and CI > 1 indicate synergy, additivity, and antagonism, respectively (Chou, 2010). For the complete DOX × NIR response matrix, Bliss-independent viability was calculated as V_Bliss_ = V_chemo_ × V_PTT_/100, and Excess Bliss was defined as V_Bliss_ − V_observed_ (Demidenko and Miller, 2019; Ianevski et al., 2022). For the representative single-dose analysis, the observed combination response was compared with the Bliss-independent expectation derived from the matched monotherapies. Interaction significance was assessed from the independent replicate data using the Demidenko-Miller framework (Demidenko and Miller, 2019). For Loewe analysis, monotherapy and fixed-ratio combination dose-response curves were fitted using the chemotherapy dose axis and the composite photothermal treatment-intensity axis. Doses corresponding to Fa = 0.25, 0.50, and 0.75 were obtained *post hoc*, and the same-Fa combination points were compared with the corresponding Loewe additivity isoboles (Chou, 2010; Ianevski et al., 2022).

### 2.7 Preliminary microfluidic perfusion evaluation

A preliminary cell-free microfluidic test was performed using a straight-channel glass-bottom slide (µ-Slide I 0.4 Luer Glass Bottom, ibidi GmbH; 50 mm × 5.0 mm × 0.45 mm; channel volume 112.5 µL) connected to a PHD ULTRA syringe pump (Harvard Apparatus) with FEP tubing (0.50 mm i.d.). For particle recovery and DLS/PDI, 1-, 2-, and 3-HMPB@mSiO_2_ (250 µg·mL^-1^) were dispersed in PBS (pH 7.4) containing 10% FBS and perfused at 5, 20, or 50 µL·min^-1^ at 37 °C; after stable flow was established, 200 µL outlet samples were collected, and particle recovery was calculated as C_out_/C_in_ × 100%, where C_out_ and C_in_ are the inlet and outlet particle concentrations determined from matched optical measurements. For DOX-loaded particles, 24 h leakage was measured using 2.0 mL of dispersion (250 µg·mL^-1^) under closed-loop circulation at the same flow rates, with released DOX quantified at 482 nm. Photothermal measurements were performed separately at 20 µL·min^-1^ using 500 µg·mL^-1^ dispersions under 808 nm irradiation (2.0 W·cm^-2^, 300 s), with temperature recorded using a FLIR ONE thermal camera. Each condition was independently repeated three times (n = 3).

### 2.8 Physicochemical characterization

Transmission electron microscopy (TEM) was performed using an FEI Tecnai G2 F20 S-Twin microscope. External particle dimensions, internal cavity dimensions, and shell thicknesses were quantified from TEM images using ImageJ (National Institutes of Health, USA). The geometric calculations used for hollow-volume estimation are provided in the Supplementary Material, and shell-thickness analysis followed the approach reported for hollow mesoporous silica TEM measurements (Šoltys et al., 2016). Nitrogen adsorption-desorption isotherms were measured at 77 K with a Quantachrome Quadrasorb SI analyzer. Specific surface area was calculated by the Brunauer-Emmett-Teller (BET) method, and pore-size distributions were obtained by the Barrett-Joyner-Halenda (BJH) method. FT-IR spectra were recorded on a Bruker VERTEX 70 spectrometer over 400–4,000 cm^-1^. XRD patterns were collected on a Bruker D8 Advance diffractometer using Cu Kα radiation (λ = 1.5406 Å). UV-vis spectra were recorded with a Shimadzu UV-3600 spectrophotometer. Hydrodynamic-size distributions and polydispersity index (PDI) were measured by dynamic light scattering. Zeta potential was measured by electrophoretic light scattering using a Zetasizer Nano ZS90 (Malvern Instruments Ltd., Malvern, UK). High-content fluorescence imaging was performed with an Operetta CLS system (PerkinElmer), and NIR temperature profiles were recorded with a FLIR ONE thermal camera.

### 2.9 Statistical analysis

Where applicable, data are presented as mean ± SD. Unless otherwise stated, multiple-group comparisons were performed by one-way ANOVA followed by Tukey’s *post hoc* test in GraphPad Prism 10. The corresponding combined-treatment groups for the three hollow-state formulations were compared directly by the same *post hoc* procedure. MFI data were analyzed by the same ANOVA/Tukey approach. For the supplementary single-dose Bliss analysis, interaction significance was evaluated from independent replicate data using the Demidenko-Miller Bliss-independence framework (Demidenko and Miller, 2019). P < 0.05 was considered statistically significant.

## 3 Results and discussion

### 3.1 Morphological characterization

Figure 2a illustrates the HCl/PVP-mediated hollowing process. The internal architecture changed systematically across the three principal etching conditions. At 0.5 M HCl, 1-HMPB retained a comparatively dense, porous interior (Figure 2b). At 0.75 M, 2-HMPB developed a distinct cavity while retaining residual PB in the central region (Figure 2c). At 1.0 M, 3-HMPB showed the most extensively hollow interior (Figure 2d). This progression is consistent with PVP-mediated surface stabilization and diffusion-controlled internal dissolution (Koczkur et al., 2015), together with a Kirkendall-type hollowing process (Yin et al., 2004). The broader etching screen defined the morphology range around these benchmark conditions. The 0.25 M sample remained comparatively dense (Supplementary Figure S1a), 0.60 M produced partial internal etching (Supplementary Figure S1b), and 0.90 M produced a highly hollow structure approaching the 1.0 M state (Supplementary Figure S1c). At 1.10 M, visible shell disruption and particle damage occurred (Supplementary Figure S1d). Independent repeat syntheses reproduced the same sequence. Batch 2 showed comparatively dense 1-HMPB, partially hollow 2-HMPB, and extensively hollow 3-HMPB (Supplementary Figure S2a–c), and Batch 3 reproduced these morphologies in Supplementary Figure S2a′–c′. These data support the reproducibility of HCl-mediated morphology control.

**FIGURE 2:**
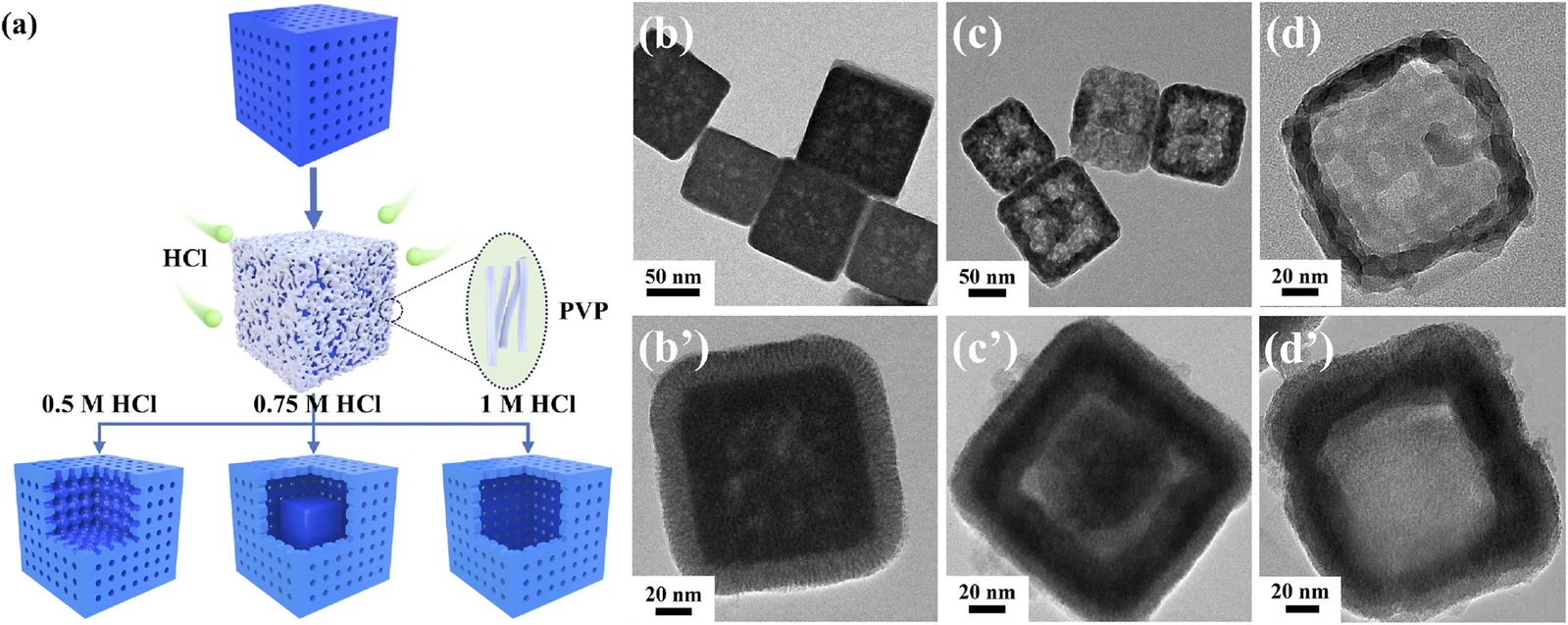
**(a)** Schematic of HCl/PVP-mediated hollowing and TEM images of **(b)** 1-HMPB, **(c)** 2-HMPB, and **(d)** 3-HMPB, **(b′-d′)** the corresponding mSiO_2_-coated nanocubes.

TEM/ImageJ analysis provided semi-quantitative structural parameters for the principal HMPB samples (Šoltys et al., 2016). A continuous closed cavity could not be reliably delineated in 1-HMPB; therefore, PB-wall thickness and hollow-volume parameters were not assigned. In 2-HMPB and 3-HMPB, PB-wall thickness decreased from 22.4 ± 2.7 to 13.4 ± 2.7 nm. Based on the TEM-derived geometric model in the Supplementary Material, the estimated hollow volumes were 1.42 ± 0.88 × 10^6^ and 1.49 ± 0.58 × 10^6^ nm^3^, respectively, whereas the estimated hollow-volume fraction increased from 33.9% ± 4.2% to 53.0% ± 2.4%. Because the absolute hollow-volume estimates overlapped substantially, PB-wall thickness and estimated hollow-volume fraction were used as the principal descriptors of hollowing. After the common mSiO_2_-coating procedure, each formulation retained its characteristic internal state. 1-HMPB@mSiO_2_ remained comparatively dense beneath the external coating (Figure 2b′), 2-HMPB@mSiO_2_ retained a partially hollow cavity with residual central PB (Figure 2c′), and 3-HMPB@mSiO_2_ retained the extensively hollow cavity (Figure 2d′). The external silica layer was most readily resolved in 1-HMPB@mSiO_2_ and had an apparent thickness of approximately 18.3 ± 0.6 nm. In 2- and 3-HMPB@mSiO_2_, the PB-wall/silica interface was less distinct in TEM projection. Independent mSiO_2_ wall thicknesses were therefore not assigned for these two formulations.

### 3.2 Physicochemical characterization

FT-IR spectra of n-HMPB@mSiO_2_ showed new bands at 1,095 and 960 cm^-1^, assigned to Si-O-Si and Si-OH vibrations, respectively (Figure 3a). The characteristic CTAB C-H stretching bands at 2,830–2,970 cm^-1^ were nearly absent after HCl-ethanol extraction, supporting silica formation and surfactant-template removal. After mSiO_2_ coating, the wide-angle XRD patterns exhibited a broad feature at approximately 2θ = 22.8°, consistent with amorphous silica (Figure 3b). All three coated samples displayed type IV N_2_ adsorption-desorption isotherms with hysteresis loops (Figure 3c), confirming mesoporosity. BET surface areas were 598, 674, and 879 m^2^·g^-1^ for 1-, 2-, and 3-HMPB@mSiO_2_, respectively. The corresponding average pore diameters were 3.2, 3.2, and 4.2 nm (Figure 3d).

**FIGURE 3:**
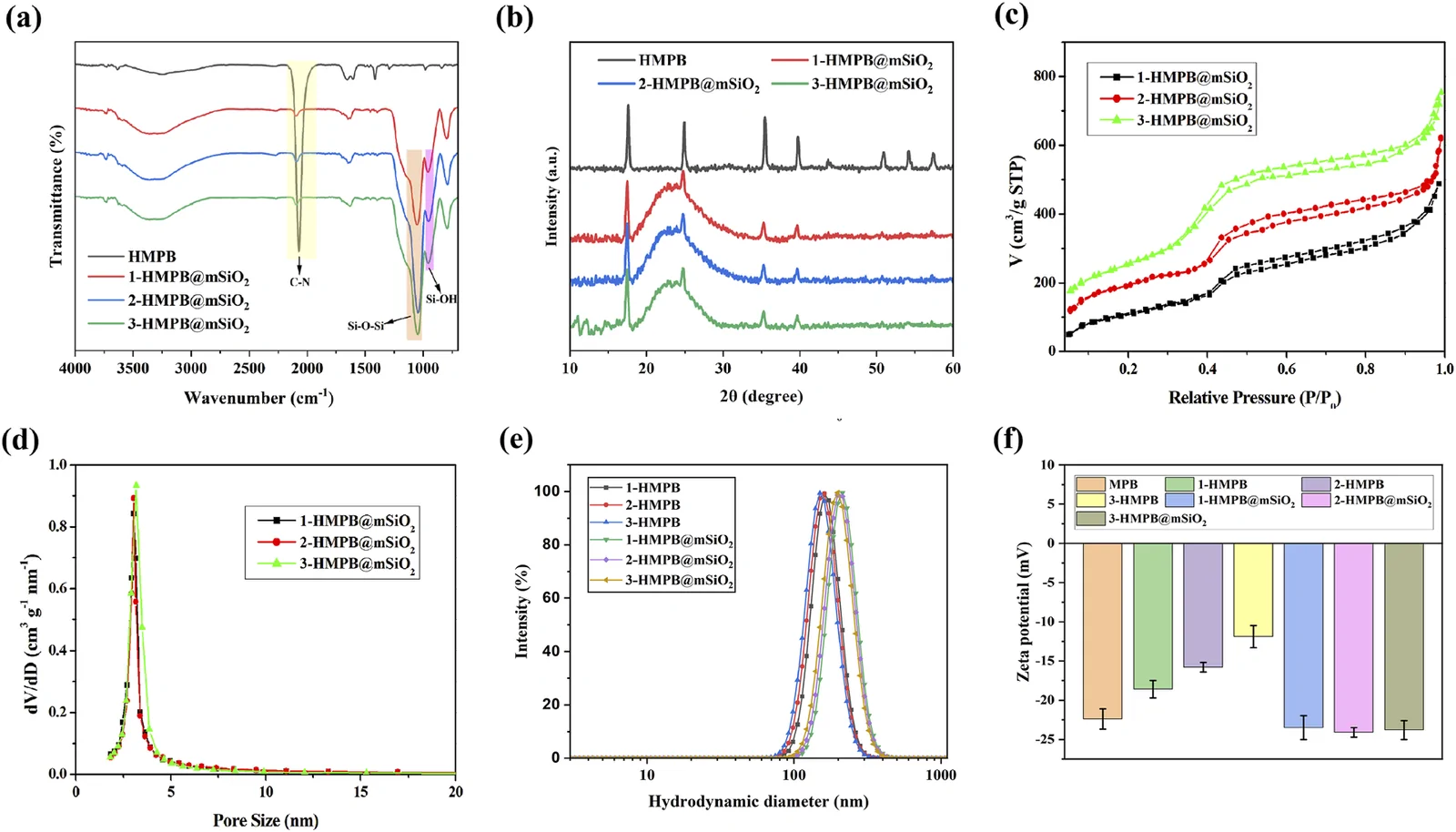
Physicochemical characterization of the HMPB@mSiO_2_ series. **(a)** FT-IR spectra of HMPB and n-HMPB@mSiO_2_; **(b)** wide-angle XRD patterns; **(c)** N_2_ adsorption-desorption isotherms of n-HMPB@mSiO_2_; **(d)** pore-size distributions; **(e)** DLS hydrodynamic-size distributions of 1-HMPB, 2-HMPB, 3-HMPB and the corresponding mSiO_2_-coated particles; and **(f)** zeta potentials of MPB, the three HMPB variants, and the corresponding mSiO_2_-coated nanoparticles in deionized water.

All six HMPB-based dispersions showed unimodal DLS intensity distributions (Figure 3e). The Z-average diameters of 1-, 2-, and 3-HMPB were 163.7 ± 4.3, 157.4 ± 3.6, and 151.6 ± 4.3 nm, with PDI values of 0.176 ± 0.013, 0.193 ± 0.011, and 0.214 ± 0.018, respectively. After mSiO_2_ coating, the corresponding diameters increased to 211.3 ± 5.5, 204.6 ± 4.7, and 197.2 ± 5.2 nm, while the PDI values were 0.164 ± 0.014, 0.181 ± 0.017, and 0.202 ± 0.020. The coating-associated diameter increases were 47.6, 47.2, and 45.6 nm, corresponding to hydrated radial increments of approximately 23.8, 23.6, and 22.8 nm. These similar increments, together with the directly resolved silica layer of 1-HMPB@mSiO_2_ in TEM, support a broadly comparable external mSiO_2_ contribution across the three formulations despite their different internal structures. Zeta potential became progressively less negative after HCl etching: MPB was −22.4 ± 1.3 mV, while 1-, 2-, and 3-HMPB were −18.6 ± 1.1, −15.8 ± 0.6, and −11.9 ± 1.4 mV, respectively (Figure 3f). After mSiO_2_ coating, the values shifted to −23.5 ± 1.5, −24.1 ± 0.6, and −23.8 ± 1.2 mV, indicating similar negatively charged outer surfaces for all three coated formulations.

### 3.3 Photothermal performance characterization

The infrared thermal images showed progressive, spatially uniform heating of all three n-HMPB@mSiO_2_ dispersions during 808 nm irradiation, whereas the water control showed only a small temperature change (Figure 4a). Figure 4b documents the irradiation and temperature-monitoring configuration. At a fixed power density of 1 W·cm^-2^, heating increased with particle concentration from 0 to 1,000 μg·mL^-1^ (Figure 4c). At a fixed concentration of 500 μg·mL^-1^, increasing the power density from 0.5 to 2.0 W·cm^-2^ also increased the temperature rise (Figure 4d). Under matched composite-particle concentrations, photothermal performance consistently followed 1-HMPB@mSiO_2_ > 2-HMPB@mSiO_2_ > 3-HMPB@mSiO_2_. All three formulations maintained similar heating-cooling profiles over three consecutive ON/OFF cycles (Figure 4e), demonstrating stable performance during repeated irradiation. Independent conversion-efficiency measurements quantified the same trend. At 200 μg·mL^-1^, A_808_ values were 0.76, 0.65, and 0.56 for 1-, 2-, and 3-HMPB@mSiO_2_, respectively (Supplementary Figure S3a). The corresponding maximum temperatures were 75.38, 65.41, and 56.84 °C (Supplementary Figure S3b), and cooling-curve analysis yielded time constants of 160.56, 171.48, and 185.28 s (Supplementary Figure S3c). The measured photothermal conversion efficiencies of 1-, 2-, and 3-HMPB@mSiO_2_ were 23.37%, 18.62%, and 14.41%, respectively.

**FIGURE 4:**
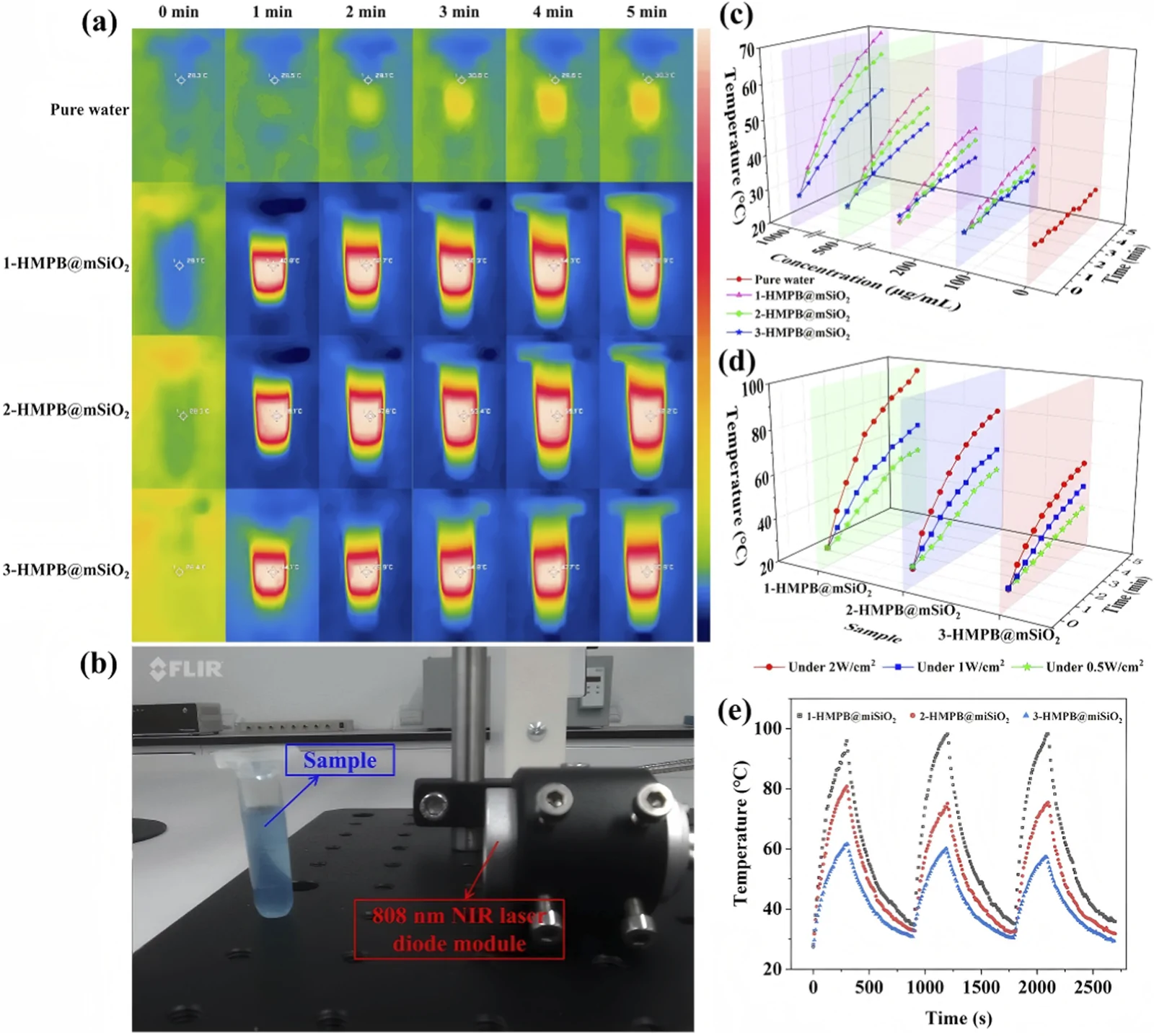
**(a)** Infrared thermal images and **(b)** experimental irradiation and temperature-monitoring setup; **(c)** temperature elevation profiles at different particle concentrations (0–1,000 μg·mL^-1^; 808 nm, 1 W·cm^-2^, 5 min); **(d)** heating curves of 500 μg·mL^-1^ n-HMPB@mSiO_2_ at different power densities (0.5–2.0 W·cm^-2^, 5 min); **(e)** three laser ON/OFF cycles of 500 μg·mL^-1^ n-HMPB@mSiO_2_ (808 nm, 2.0 W·cm^-2^, 5 min irradiation followed by 10 min cooling).

Because the initial measurements were performed at the same composite concentration, the contribution of PB abundance was further examined by ICP-MS and PB-equivalent-mass-matched photothermal measurements. The Fe contents of 1-, 2-, and 3-HMPB@mSiO2 were 31.40 ± 0.80, 26.60 ± 0.70, and 21.30 ± 0.60 wt%, corresponding to PB-equivalent mass fractions of 69.01%, 58.46%, and 46.81%, respectively (Supplementary Table S2). When the three formulations were experimentally matched to the same PB-equivalent mass, their photothermal conversion efficiencies converged to 16.80%, 15.80%, and 14.41%, compared with 23.37%, 18.62%, and 14.41% at equal composite concentration. This marked reduction in the difference indicates that PB abundance is a major contributor to the hollow-state-dependent photothermal response, while the residual trend suggests an additional contribution from structural and interfacial factors.

### 3.4 Drug loading and release characterization

The DOX calibration curve was linear over the tested concentration range (A = 0.0055C + 0.0026, R = 0.99995; Figure 5a) and was used to quantify residual unloaded DOX. The combined supernatant and washing solutions showed progressively lower residual DOX signals from 1-HMPB@mSiO_2_ to 2-HMPB@mSiO_2_ and 3-HMPB@mSiO_2_ (Figure 5b), consistent with greater drug uptake by the more hollow carriers. In the main loading experiment, DLC values were 525, 714, and 867 μg·mg^-1^ and EE values were 52.5%, 71.4%, and 86.7% for 1-, 2-, and 3-HMPB@mSiO_2_, respectively (Table 1). Two additional independently prepared batches were analyzed to assess batch-to-batch reproducibility. Across the three batches, mean DLC values were 551.3 ± 23.5, 693.0 ± 19.3, and 896.7 ± 26.5 μg·mg^-1^, with CV values of 4.3%, 2.8%, and 3.0%, respectively (Supplementary Table S3). The low CVs and unchanged loading rank support reproducible drug-loading performance across batches.

**FIGURE 5:**
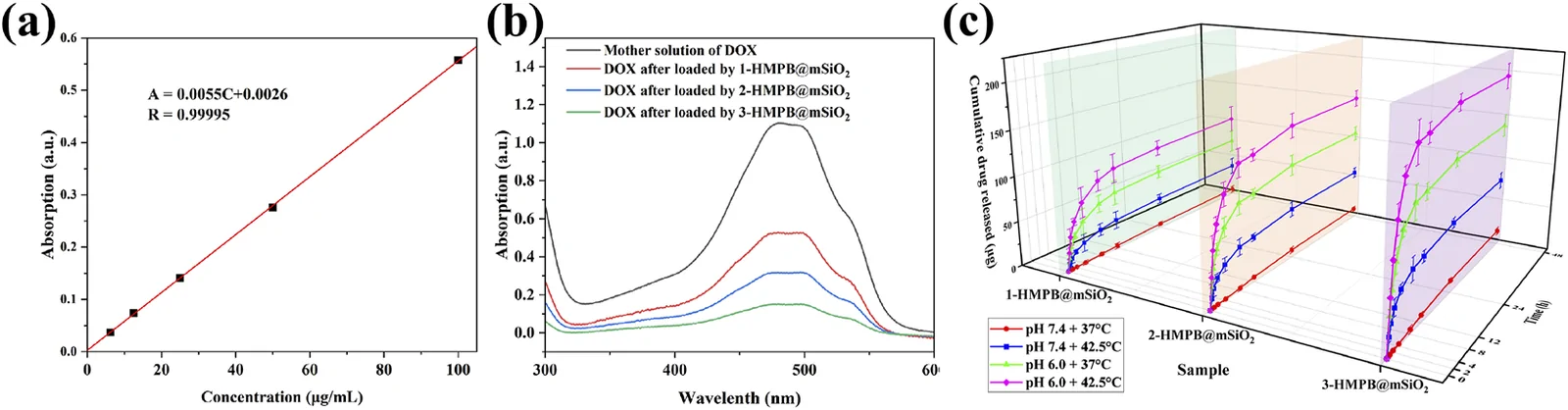
**(a)** DOX calibration curve; **(b)** UV-vis spectra of DOX before and after loading by 1-, 2-, and 3-HMPB@mSiO_2_; **(c)** time-dependent DOX release under pH 7.4/37 °C, pH 7.4/42.5 °C, pH 6.0/37 °C, and pH 6.0/42.5 °C.

**TABLE 1:** Drug loading results of n-HMPB@mSiO_2_.

Sample	Residual mother liquor of DOX in PBS solution			Drug-loading performance		
	A_unloaded_ ^a^	C_unloaded_ ^b^ (μg·mL^-1^)	W_unloaded_ ^c^ (μg)	W_loaded_ ^d^ (μg)	DLC^e^ (μg·mg^-1^)	EE^f^ (%)
1-HMPB@mSiO_2_	0.525	95.0	2,375	2,625	525	52.5
2-HMPB@mSiO_2_	0.317	57.2	1,430	3,570	714	71.4
3-HMPB@mSiO_2_	0.149	26.6	665	4,335	867	86.7

a A_unloaded_: Absorbance of unloaded DOX, in PBS, solution.

b C_unloaded_: Concentration of unloaded DOX, in PBS, solution.

c W_unloaded_: Weight of unloaded DOX.

d W_loaded_: Weight of DOX, successfully loaded.

e DLC: Weight-based drug loading Content.

f EE: encapsulation efficiency.

DOX release depended on both environmental condition and carrier hollow state (Figure 5c). For every carrier, release was lowest at pH 7.4/37 °C. Lowering the pH to 6.0 at 37 °C or raising the temperature to 42.5 °C at pH 7.4 increased release. The combined pH 6.0/42.5 °C condition produced the greatest release. Across all four conditions, the released amount followed the order 3-HMPB@mSiO_2_ > 2-HMPB@mSiO_2_ > 1-HMPB@mSiO_2_. Thus, mild acidity and mild hyperthermia each promoted DOX release, while greater hollowing increased the amount of drug released.

### 3.5 *In vitro* cellular performance and quantitative uptake

Treatment-matched cells + nanoparticle/no-MTT controls showed increasing cell-associated optical background with nanoparticle concentration (Supplementary Figure S4a); this treatment-matched term was directly subtracted from the corresponding sample signal before viability normalization. By contrast, the separate cell-free nanoparticle + MTT controls produced only small residual signals after the same analytical workflow (Supplementary Figure S4b). The cell-free result therefore served as an independent check that direct nanoparticle-MTT reactivity and residual particulate absorbance made only a minor contribution after processing; it was not subtracted a second time, which would duplicate the nanoparticle-background correction already performed in the cell-containing wells. After the treatment-matched correction, all three blank carriers maintained high 24 h HeLa-cell viability across 62.5–1,000 μg·mL^-1^ (Figure 6a). Even at 1,000 μg·mL^-1^, corrected viability remained approximately 82%–84%, indicating limited acute carrier-associated cytotoxicity under the tested conditions.

**FIGURE 6:**
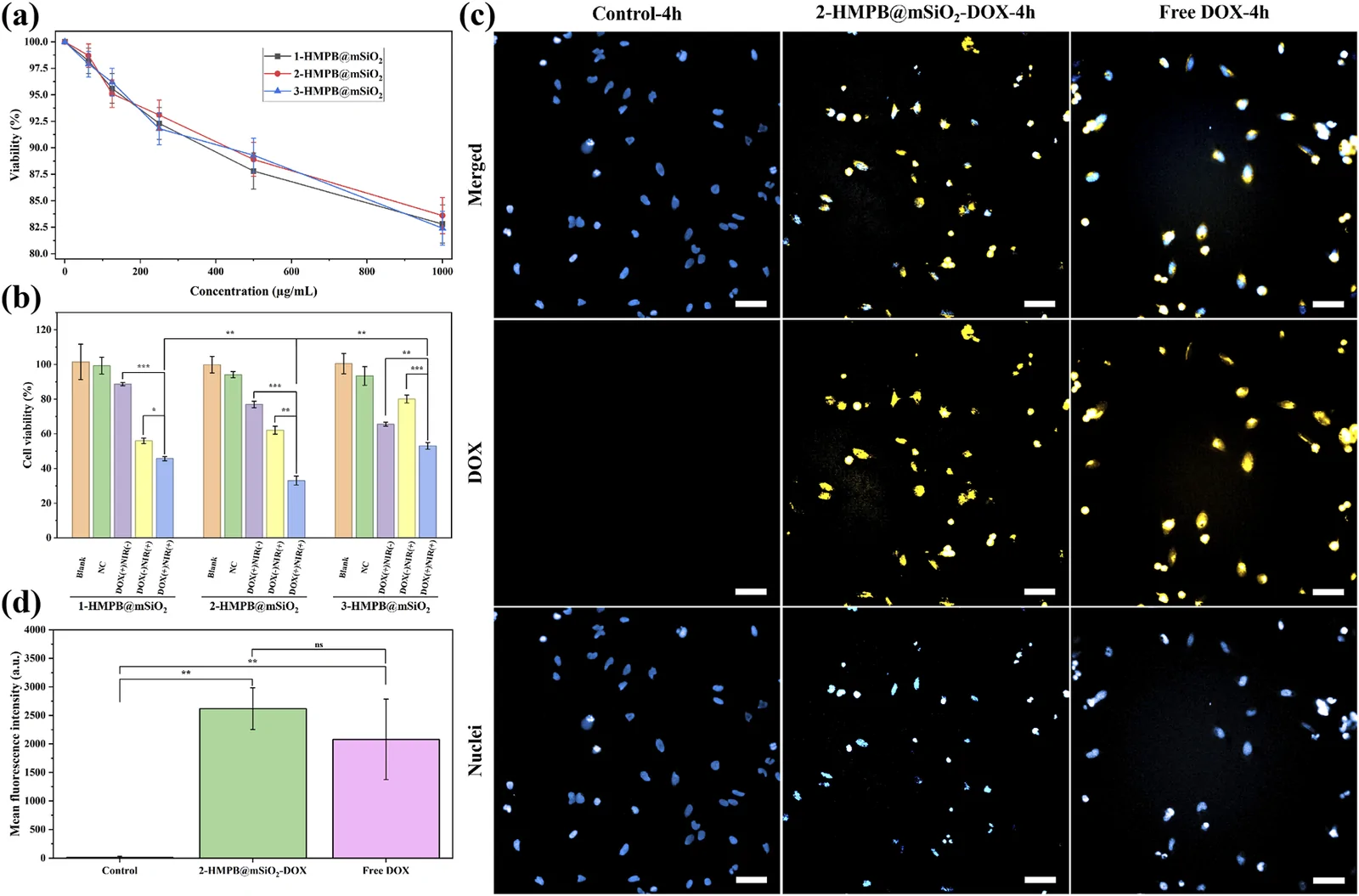
**(a)** Treatment-matched nanoparticle-interference-corrected viability of HeLa cells exposed to n-HMPB@mSiO_2_; **(b)** therapeutic cytotoxicity under carrier, chemotherapy, PTT, and combined-treatment conditions; **(c)** high-content images after 4 h treatment with control medium, 2-HMPB@mSiO_2_-DOX, or free DOX; and **(d)** quantitative DOX mean fluorescence intensity (MFI). Data are mean ± SD; statistical significance is indicated in the figure.

Therapeutic response varied with hollow structure (Figure 6b). In the chemotherapy-only groups, 3-HMPB@mSiO_2_-DOX produced the strongest cytotoxicity, consistent with its higher drug loading and release. In the PTT-only groups, 1-HMPB@mSiO_2_ produced the strongest response, consistent with its higher photothermal conversion. Under combined treatment, 2-HMPB@mSiO_2_-DOX yielded the lowest HeLa-cell viability, approximately 33%. ANOVA/Tukey comparisons confirmed that viability in the 2-HMPB@mSiO_2_-DOX combination group was significantly lower than in the corresponding 1-HMPB@mSiO_2_-DOX and 3-HMPB@mSiO_2_-DOX combination groups (P < 0.01).

High-content imaging showed strong DOX fluorescence after treatment with either 2-HMPB@mSiO_2_-DOX or free DOX, with substantial overlap with the nuclear signal (Figure 6c). Quantitative MFI was significantly higher in both DOX-containing groups than in the untreated control (P < 0.01), whereas 2-HMPB@mSiO_2_-DOX and free DOX did not differ significantly (Figure 6d). These imaging data support efficient intracellular delivery of DOX by the nanocarrier.

Because the cellular evaluation was limited to HeLa cells, future studies should include additional cancer cell lines, normal/non-malignant cells, three-dimensional tumor models, and *in vivo* systems to further assess therapeutic generalizability and biosafety.

### 3.6 Quantitative chemo-photothermal synergy analysis

Because nanoparticle-mediated PTT depends jointly on particle concentration and NIR exposure, the PTT dose used for CI and Loewe analysis represents the predefined composite treatment-intensity factor rather than NIR fluence alone. Figure 7a shows dose-dependent decreases in viability for chemotherapy alone and PTT alone, with a stronger response for the matched combination across the middle and high dose range. For the fixed-ratio series, the CI values were 1.098, 0.959, 0.857, 0.789, 0.744, 0.710, and 0.656 at Fa = 0.077, 0.242, 0.426, 0.577, 0.684, 0.759, and 0.852, respectively (Figure 7b). The resulting CI-Fa trend therefore shifts from near-additive/slightly antagonistic behavior at the lowest effect level to progressively stronger synergy as the affected fraction increases. A plausible explanation is that, at low exposure, both chemotherapy and photothermal stress remain submaximal and the CI estimate is more sensitive to small deviations in the monotherapy dose-effect fits; as the dose rises, stronger photothermal stress can facilitate drug release and increase cellular sensitivity to chemotherapy, allowing the combined effect to become increasingly synergistic (Chou, 2010; Yuan et al., 2016). Across the complete 8 × 8 response matrix, positive Excess Bliss values covered a broad combination region and reached 17.0 percentage points at 156.3 μg·mL^-1^ DOX equivalent plus 300 J·cm^-2^ NIR (Figure 7c). In the *post hoc* Loewe analysis using the composite photothermal treatment-intensity axis, same-Fa combination points at Fa = 0.25, 0.50, and 0.75 fell below the corresponding additivity isoboles, with CI values of approximately 0.95, 0.82, and 0.71, respectively (Supplementary Figure S5b) (Chou, 2010; Demidenko and Miller, 2019; Ianevski et al., 2022). Together, the dose-dependent CI trend, Bliss response surface, and Loewe analysis support a progressively stronger chemo-photothermal interaction for the intermediate-hollow formulation.

**FIGURE 7:**
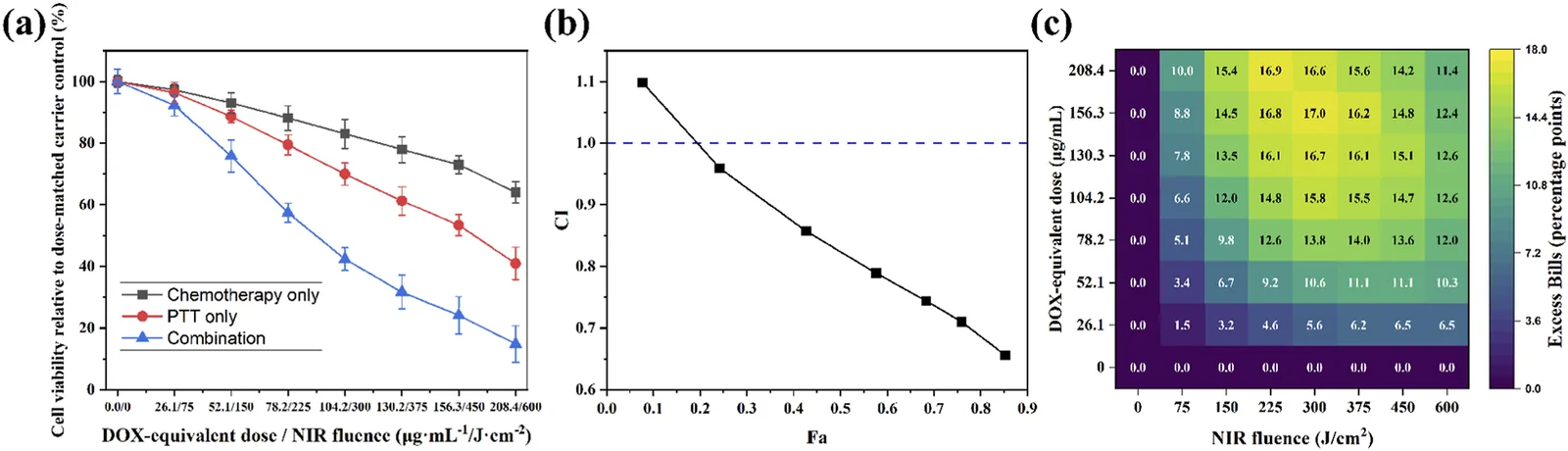
Quantitative chemo-photothermal interaction analysis for 2-HMPB@mSiO_2_-DOX. **(a)** Carrier-normalized dose-response curves for chemotherapy only, PTT only, and the matched combination; **(b)** combination index (CI) *versus* affected fraction (Fa) for the experimentally tested fixed-ratio dose levels; and **(c)** Excess Bliss heatmap across the complete DOX-equivalent dose × NIR-fluence matrix. Positive Excess Bliss values indicate viability lower than the Bliss-independent expectation.

### 3.7 Preliminary microfluidic perfusion evaluation

Figure 8a shows the syringe-pump-driven microfluidic configuration used to examine particle transport and NIR response in physiological medium. Particle recovery remained high at 5, 20, and 50 μL·min^-1^ but declined moderately as flow rate increased, with the largest decrease observed for 3-HMPB@mSiO_2_ (Figure 8b). The pre-perfusion Z-average diameters measured in physiological medium were modestly larger than the water-based DLS Z-average diameters in Figure 3e, consistent with a medium-dependent change in the particle interfacial environment. After perfusion, Z-average diameter increased only modestly relative to the corresponding physiological-medium inlet value (Figure 8c), and PDI remained within a narrow range across the tested flow rates (Figure 8d). These observations provide preliminary evidence of acceptable colloidal stability during short-term perfusion in physiological medium. The 24 h DOX leakage under flow remained limited and retained the structure-dependent order 3-HMPB@mSiO_2_ > 2-HMPB@mSiO_2_ > 1-HMPB@mSiO_2_ (Figure 8e). At 20 μL·min^-1^, NIR heating under physiological flow reproduced the static photothermal ranking, with 1-HMPB@mSiO_2_ showing the greatest temperature rise and 3-HMPB@mSiO_2_ the lowest (Figure 8f). Thus, the principal structure-dependent trends in transport, dispersion, drug retention, and photothermal response were retained under the tested physiological-medium flow conditions. Future studies should assess longer-term colloidal stability and performance in cell-containing tumor-chip and vascularized microphysiological models.

**FIGURE 8:**
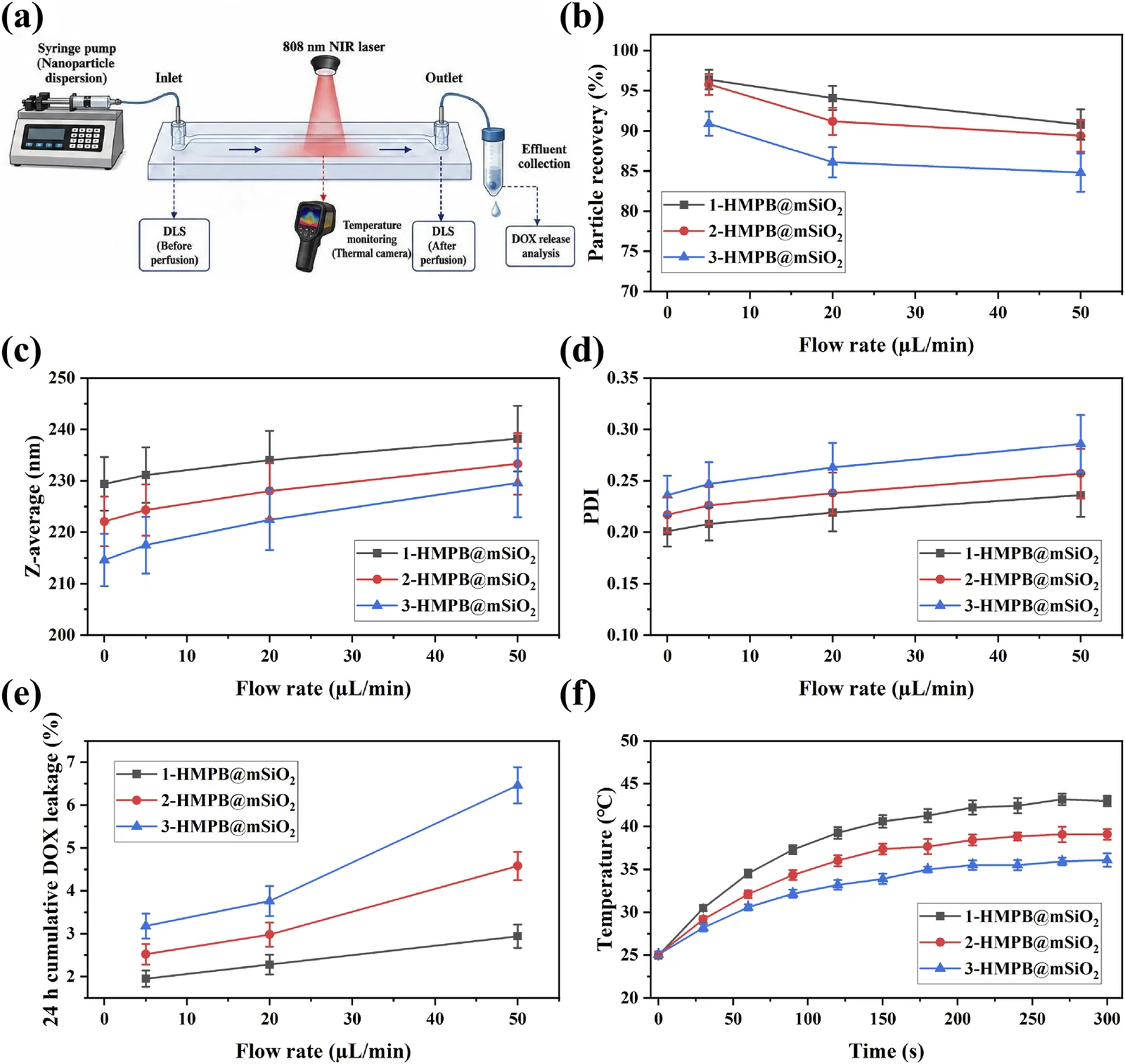
Preliminary microfluidic perfusion evaluation. **(a)** Experimental flow and irradiation configuration; **(b)** particle recovery; **(c)** Z-average diameter measured in physiological medium; **(d)** PDI; **(e)** 24 h cumulative DOX leakage; and **(f)** NIR-induced temperature profiles at 20 μL·min^-1^.

## 4 Conclusion

Controlled HCl etching generated three distinct HMPB hollow states and revealed an inverse relationship between PB-dependent photothermal conversion and hollow-state-associated drug loading/release. Low-hollow 1-HMPB@mSiO_2_ showed the strongest photothermal response, whereas high-hollow 3-HMPB@mSiO_2_ provided the greatest DOX loading and release. Intermediate 2-HMPB@mSiO_2_-DOX produced the most favorable combined-treatment response at the equal total formulation concentration. Multi-dose CI, Bliss, and Loewe analyses supported increasingly synergistic interactions at moderate-to-high effect levels, with near-additive/slightly antagonistic behavior at the lowest effect level. The broader etching screen, ImageJ-derived structural parameters, and independent repeat-batch TEM images supported structural differentiation and reproducibility. Preliminary microfluidic perfusion preserved the main structure-dependent trends in dispersion, drug leakage, and NIR heating under flow. Further work should address additional malignant and non-malignant cell models, microphysiological tumor systems, long-term biosafety and biodegradation, pharmacokinetics/biodistribution and clearance (Qu et al., 2023; Dogra et al., 2018; Zhang et al., 2024), and process-scale reproducibility and quality control (Noureddine et al., 2023).

## Data Availability

Data are available from the corresponding authors upon reasonable request. The raw data supporting the conclusions of this article will be made available by the authors, without undue reservation.
