# Supplementary material for: Hollow-degree-tuned Prussian blue–mesoporous silica nanoplatforms for chemo-photothermal cancer therapy with preliminary microfluidic perfusion evaluation
Source: Front Bioeng Biotechnol. 2026 Sep 14;14:1932857. doi: 10.3389/fbioe.2026.1932857 (PMC13617051; doi:10.3389/fbioe.2026.1932857)
Supplement: Supplementary file 1 [file Supplementaryfile1.docx]

***Supplementary Material***

**Hollow-Degree-Tuned Prussian Blue–Mesoporous Silica Nanoplatforms for Chemo-Photothermal Cancer Therapy with Preliminary Microfluidic Perfusion Evaluation**

Jingjing Xi^1,2^, Jingjing Liu^1^, Heyao Zhang^1^, Mengxi Wang^1^, Chenyi Lu^1^, Tinghan Wang^1^, Xijun Piao^3^, Ri Huang^3^, Keyun Ren^3*^, Xiao Mei^1,3*^

^1^ Center of Self-Propelled Nanotechnologies, Suzhou Industrial Park Institute of Services Outsourcing, Suzhou, China

^2^ Suzhou Huitong Chromatographic Separation and Purification Co., Ltd., Suzhou, China

^3^ Catug Biotechnology (Suzhou) Co., Ltd., Suzhou, China

* Correspondence: Xiao Mei, meix@siso.edu.cn; Keyun Ren, keyun.ren@catugbio.com

**Table of Contents**

TEM-Based Hollow-Structure Analysis	3

Photothermal Conversion Efficiency	6

PB-Equivalent-Mass-Matched Photothermal Measurements	8

Batch-to-Batch Variation in DOX-Loading Performance	10

MTT Optical-Interference Analysis	11

Chemo-Photothermal Synergy Analysis	13

References	15

# **TEM-Based Hollow-Structure Analysis**

TEM images were analyzed using ImageJ (National Institutes of Health, USA). External PB dimensions and internal low-electron-density cavity dimensions were converted to equivalent square edge lengths from the corresponding projected areas, following ImageJ-based shell-thickness measurement of hollow mesoporous silica particles [1]. For particles with a clearly resolved cavity, the PB wall thickness was calculated as:

$$t_{\mathrm{PB}}=\frac{L_{\mathrm{PB}}-L_{\mathrm{cav}}}{2}$$

where t_PB_ is the PB-wall thickness, L_PB_ is the equivalent outer PB-core edge length, and L_cav_ is the equivalent cavity edge length.

For 2-HMPB, discrete residual PB domains within the cavity were represented as equivalent spheres using their measured projected areas. The equivalent diameter and total residual-PB volume were calculated as:

$$d_{res,i}=2\sqrt{\frac{A_{res,i}}{\pi}}$$

where d_res,i_ is the equivalent diameter of residual PB domain i, A_res,i_ is its projected TEM area, and i indexes the discrete residual PB domains.

$$V_{\mathrm{res}}=\sum_{i} \frac{\pi d_{res,i}^{3}}{6}$$

where V_res_ is the summed equivalent volume of all residual PB domains and d_res,i_ is the equivalent diameter of residual domain i.

Assuming approximately cubic external and cavity geometries, the estimated hollow volume and hollow-volume fraction were calculated as:

$$V_{\mathrm{hollow}}=L_{\mathrm{cav}}^{3}-V_{\mathrm{res}}$$

where V_hollow_ is the estimated hollow volume, L_cav_ is the equivalent cavity edge length, and V_res_ is the residual-PB volume within the cavity.

$$\phi_{\mathrm{hollow}}=\frac{V_{\mathrm{hollow}}}{L_{\mathrm{PB}}^{3}}\times100\%$$

where ϕ_hollow_ is the hollow-volume fraction, V_hollow_ is the estimated hollow volume, and L_PB_ is the equivalent outer PB-core edge length.

For 3-HMPB, no distinct residual central PB domain was resolved and V_res_ was taken as zero. For 1-HMPB, a continuous closed cavity boundary could not be reliably delineated; therefore, PB wall thickness and hollow-volume parameters were reported as not determined.

**Figure S1.** Representative TEM images of PB nanocubes obtained after etching at additional HCl concentrations: (a) 0.25 M, (b) 0.60 M, (c) 0.90 M, and (d) 1.10 M.

**Figure S2.** TEM images from two additional independent synthesis batches. Figure S2(a–c) shows 1-HMPB, 2-HMPB, and 3-HMPB from Batch 2, respectively; Figure S2(a′–c′) shows the corresponding samples from Batch 3. Main-text Figure 2 contains the Batch 1 TEM images.

# **Photothermal Conversion Efficiency**

Photothermal conversion efficiency (η) was calculated from the independently measured absorbance, heating, cooling, and water-blank data using the method adapted from Hoffman et al. [2]. The cooling time constant (τ_s_) was obtained from the linear relationship between ln(T − T_amb_) and cooling time using the same 60-480 s fitting interval for all samples:

$$\ln\left( T-T_{\mathrm{amb}} \right)=-\frac{t}{\tau_{s}}+C$$

where T is the sample temperature at cooling time t, T_amb_ is the ambient temperature, τ_s_ is the characteristic cooling time constant, and C is the regression intercept.

The heat-transfer coefficient-area product was obtained from:

$$hS=\frac{mC_{p}}{\tau_{s}}$$

where h is the heat-transfer coefficient, S is the effective heat-transfer area, m is the mass of the dispersion, C_p_ is its effective specific heat capacity, and τ_s_ is the cooling time constant.

Photothermal conversion efficiency was then calculated as:

$$\eta=\frac{hS\left( T_{\max}-T_{\mathrm{amb}} \right)-Q_{0}}{I\left( 1-{10}^{-A_{808}} \right)}$$

where η is the photothermal conversion efficiency, hS is the heat-transfer coefficient-area product, T_max_ is the maximum sample temperature, T_amb_ is the ambient temperature, Q_0_ is the water/container background heat contribution, I is the actual incident laser power, and A_808_ is the absorbance at 808 nm.

**Figure S3.** Measurements used for photothermal conversion-efficiency calculation: (a) UV-vis spectra of 200 μg·mL^-1^ n-HMPB@mSiO_2_ dispersions; (b) independent 808 nm heating/cooling profiles for water and the three formulations; and (c) linearized cooling data used to obtain τ_s_.

**Table S1.** Photothermal-conversion parameters calculated from the data in Figure S3.

| **Sample** | A_808_ | T_max_ (°C) | Cooling τ_s_ (s) | **η (%)** |
| --- | --- | --- | --- | --- |
| 1-HMPB@mSiO_2_ | 0.76 | 75.38 | 160.56 | 23.37 |
| 2-HMPB@mSiO_2_ | 0.65 | 65.41 | 171.48 | 18.62 |
| 3-HMPB@mSiO_2_ | 0.56 | 56.84 | 185.28 | 14.41 |

# **PB-Equivalent-Mass-Matched Photothermal Measurements**

ICP-MS-derived Fe contents were used to determine the composite concentrations required for experimental photothermal comparison at matched PB-equivalent mass. The measured Fe contents of 1-, 2-, and 3-HMPB@mSiO_2_ were 31.40 ± 0.80, 26.60 ± 0.70, and 21.30 ± 0.60 wt% (mean ± SD, n = 3 independent digestions), respectively.

The PB-equivalent mass fraction was estimated using the theoretical Fe mass fraction of anhydrous Prussian blue, Fe_4_[Fe(CN)_6_]_3_ (0.4550):

$$w_{PB,eq}=\frac{w_{\mathrm{Fe}}}{0.4550}$$

where *w*_PB,eq_ is the calculated PB-equivalent mass fraction and *w*_Fe_ is the ICP-MS-measured Fe mass fraction. The resulting PB-equivalent mass fractions were 69.01%, 58.46%, and 46.81% for 1-, 2-, and 3-HMPB@mSiO_2_, respectively. These values were used for compositional matching and do not imply that the PB phase is perfectly anhydrous or stoichiometric.

The PB-equivalent concentration in the original 200 μg·mL^−1^ 3-HMPB@mSiO_2_ dispersion was selected as the common reference:

$$C_{PB,ref}=200\times0.4681=93.62 \mu g\cdot\mathrm{mL}^{-1}$$

For each formulation, the composite concentration required to provide the same PB-equivalent concentration was calculated as:

$$C_{comp,i}=\frac{C_{PB,ref}}{w_{PB,eq,i}}$$

Accordingly, the required composite concentrations were 135.7, 160.2, and 200.0 μg·mL^−1^ for 1-, 2-, and 3-HMPB@mSiO_2_, respectively. At a sample volume of 200 μL, these concentrations corresponded to a target PB-equivalent mass of approximately 18.73 μg per sample.

The PB-equivalent-mass-matched dispersions were subjected to photothermal conversion-efficiency measurements using the same procedure as the equal-composite-concentration samples. Each 200 μL dispersion was irradiated vertically with an 808 nm laser at an actual incident power of 1.0 W (2.5 W·cm^−2^) for 600 s, followed by 600 s of natural cooling. The absorbance at 808 nm was independently measured for each PB-equivalent-mass-matched dispersion. The same 60–480 s cooling interval and water-blank correction were used for all formulations. The cooling time constant was obtained from:

$$ln(T-T_{\mathrm{amb}})=-\frac{t}{\tau_{s}}+C$$

The heat-transfer coefficient-area product was obtained from:

$$hS=\frac{mC_{p}}{\tau_{s}}$$

Photothermal conversion efficiency was calculated as:

$$\eta=\frac{hS(T_{\max}-T_{\mathrm{amb}})-Q_{0}}{I(1-{10}^{-A_{808}})}$$

where η is the photothermal conversion efficiency, hS is the heat-transfer coefficient-area product, *T*_max_ is the maximum sample temperature, *T*_amb_ is the ambient temperature, *Q*_0_ is the water/container background heat contribution, *I* is the actual incident laser power, and *A*_808_ is the independently measured absorbance at 808 nm.

**Table S2. Photothermal parameters of n-HMPB@mSiO_2_ under PB-equivalent-mass-matched conditions.**

| **Sample** | **Fe (wt%, mean ± SD)** | **PB-eq. (wt%)** | **Comp. (μg·mL^−1^)** | **Target *m*_PB-eq._ (μg)** | ***A*_808_** | ***T*_max_ (°C)** | ***τ*_s_ (s)** | **η (%)** |
| --- | --- | --- | --- | --- | --- | --- | --- | --- |
| 1-HMPB@mSiO_2_ | 31.40 ± 0.80 | 69.01 | 135.7 | 18.73 | 0.516 | 57.83 | 172.86 | 16.80 |
| 2-HMPB@mSiO_2_ | 26.60 ± 0.70 | 58.46 | 160.2 | 18.73 | 0.521 | 57.16 | 178.25 | 15.80 |
| 3-HMPB@mSiO_2_ | 21.30 ± 0.60 | 46.81 | 200.0 | 18.73 | 0.560 | 56.84 | 185.28 | 14.41 |

# **Batch-to-Batch Variation in DOX-Loading Performance**

To assess batch-to-batch variation in drug-loading performance, drug-loading content (DLC) and encapsulation efficiency (EE) were determined independently for three n-HMPB@mSiO_2_ batches. Batch 1 corresponds to the formulation quantified in the main drug-loading experiment, whereas Batches 2 and 3 were independently prepared and loaded with DOX using the same procedure. For each formulation, the three batch-specific DLC values were summarized as mean ± SD, and batch-to-batch variability was expressed as the coefficient of variation (CV). The corresponding EE values were summarized as mean ± SD.

$$CV (\%) =\frac{SD}{Mean}\times100\%$$

where CV is the coefficient of variation, SD is the sample standard deviation of the three batch-specific DLC values, and Mean is their arithmetic mean.

**Table S3.** Batch-to-batch variation in DOX-loading performance of n-HMPB@mSiO_2_ across three independently prepared batches.

| **Sample** | DLC (μg·mg^-1^) | | | **DLC mean ± SD**  (μg·mg^-1^) | **EE (%)** | **CV (%)** |
| --- | --- | --- | --- | --- | --- | --- |
|  | **Batch 1** | **Batch 2** | **Batch 3** |  |  |  |
| 1-HMPB@mSiO_2_ | 525 | 559 | 570 | 551.3 ± 23.5 | 55.1 ± 2.3 | 4.3 |
| 2-HMPB@mSiO_2_ | 714 | 676 | 689 | 693.0 ± 19.3 | 69.3 ± 1.9 | 2.8 |
| 3-HMPB@mSiO_2_ | 867 | 905 | 918 | 896.7 ± 26.5 | 89.7 ± 2.7 | 3.0 |

# **MTT Optical-Interference Analysis**

All optical-density values were measured at 570 nm. Two complementary controls were used to address nanoparticle-related MTT interference. First, the treatment-matched cells + nanoparticle/no-MTT well quantified the cell-associated optical background remaining after nanoparticle exposure, washing, DMSO treatment, clarification, and supernatant transfer. This treatment-matched background was directly subtracted from the corresponding MTT-containing sample signal [3-4].

$${OD}_{570,corr} = {OD}_{570,sample} - {OD}_{570,cells+NP,noMTT}$$

where OD_570,corr_ is the treatment-matched corrected sample signal, OD_570,sample_ is the measured absorbance of the MTT-containing cell sample, and OD_570,cells+NP,noMTT_ is the absorbance of the matched cells + nanoparticle/no-MTT control.

Cell viability was then calculated from the corrected signal as:

$$Cell viability (\%) = {OD}_{570,corr} / ({OD}_{570,control} - {OD}_{570,cells,noNP,noMTT}) \times100$$

where OD_570,control_ is the MTT-containing untreated-cell control and OD_570,cells,noNP,noMTT_ is the untreated cells/no-nanoparticle/no-MTT optical background.

For visualization of the treatment-matched optical contribution, the cell-associated nanoparticle background increment was reported as:

$${\Delta OD}_{570,cell-associated} = {OD}_{570,cells+NP,noMTT} - {OD}_{570,cells,noNP,noMTT}$$

where ΔOD_570,cell-associated_ is the concentration-dependent optical background attributable to nanoparticle exposure in the cell-containing workflow.

Second, a separate cell-free nanoparticle + MTT control was processed in parallel to test direct nanoparticle-MTT reactivity and residual particulate interference:

$${\Delta OD}_{570,residual} = {OD}_{570,cell-free NP+MTT} - {OD}_{570,MTT blank}$$

where ΔOD_570,residual_ is the net cell-free nanoparticle + MTT signal after subtraction of the MTT reagent blank.

To relate this independent control to the final assay reliability, the residual-interference fraction was expressed relative to the corrected untreated-cell MTT signal:

$$R_{\mathrm{residual}} (\%) = {\Delta OD}_{570,residual} / ({OD}_{570,control} - {OD}_{570,cells,noNP,noMTT}) \times100$$

where R_residual_ is the residual cell-free interference fraction. ΔOD_570,residual_ was not subtracted a second time from the viability numerator because the treatment-matched cells + nanoparticle/no-MTT correction already removes the nanoparticle-associated optical contribution in the cell-containing wells; instead, the cell-free control provides an independent diagnostic of direct nanoparticle-MTT reactivity and residual particulate interference. The small residual signal in Figure S4(b), together with the treatment-matched subtraction in Figure S4(a), supports the reliability of the corrected MTT viability values.

**Figure S4.** MTT optical-interference controls: (a) treatment-matched cell-associated background, ΔOD_570,cell-associated_, which was directly incorporated into the viability correction; and (b) residual cell-free nanoparticle + MTT signal, ΔOD_570,residual_, used as an independent diagnostic of direct nanoparticle-MTT and residual particulate interference.

# **Chemo-Photothermal Synergy Analysis**

Within each independent experiment, treatment viability was normalized to the corresponding dose-matched unloaded-carrier control before calculating the final mean ± SD:

$$V_{\mathrm{norm}}\left( \% \right)=\frac{V_{\mathrm{treatment}}}{V_{dose-matched carrier}}\times100$$

where V_norm_ is carrier-normalized viability, V_treatment_ is the treatment-group viability, and V_dose-matched carrier_ is the unloaded-carrier viability at the same nanoparticle dose.

For the fixed-ratio combination series, PTT dose was represented by a dimensionless photothermal treatment-intensity factor (x) that simultaneously scaled nanoparticle concentration and NIR fluence at a fixed ratio; 1.0× corresponded to 250 μg·mL^-1^ nanoparticles and 300 J·cm^-2^. Chemotherapy dose was represented by DOX-equivalent concentration, and both treatment axes were scaled by the same x factor. The affected fraction was calculated as:

$$F_{a}=1-\frac{V_{\mathrm{combination}}}{100}$$

where F_a_ is the affected fraction and V_combination_ is the carrier-normalized combination-treatment viability expressed as a percentage.

Combination index values were obtained from the dose-effect relationships using the Chou-Talalay framework [5]. For two treatment components, the dose-sum representation is:

$$CI=\frac{D_{1}}{D_{x,1}}+\frac{D_{2}}{D_{x,2}}$$

where CI is the combination index, D_1_ and D_2_ are the two treatment-component doses in combination, and D_x,1_ and D_x,2_ are the corresponding monotherapy doses required to produce the same effect level.

Bliss-independent expected viability and Excess Bliss were calculated as [6-7]:

$$V_{\mathrm{Bliss}}=\frac{V_{\mathrm{chemo}}V_{\mathrm{PTT}}}{100}$$

where V_Bliss_ is the Bliss-independent expected viability, V_chemo_ is the chemotherapy-only viability, and V_PTT_ is the PTT-only viability, all expressed as percentages.

$$Excess Bliss=V_{\mathrm{Bliss}}-V_{\mathrm{observed}}$$

where Excess Bliss is the difference between the Bliss-expected and observed combination viabilities, and V_observed_ is the measured combination viability.

For the representative single-dose analysis, interaction significance was evaluated from the underlying independent replicate data using the Demidenko-Miller Bliss framework [6]. For Loewe analysis, monotherapy dose-response curves and the fixed-ratio combination curve were fitted; the monotherapy doses and combination points at F_a_ = 0.25, 0.50, and 0.75 were obtained post hoc and compared with the corresponding additivity isoboles:

$$\frac{D_{1}}{D_{x,1}\left( F_{a} \right)}+\frac{D_{2}}{D_{x,2}\left( F_{a} \right)}=1$$

where D_1_ and D_2_ are the combination doses at a given F_a_, and D_x,1_(F_a_) and D_x,2_(F_a_) are the monotherapy doses producing the same affected fraction; a dose sum below 1 indicates a point below the Loewe additivity isobole.

Figure S5. Chemo-photothermal synergy analyses for 2-HMPB@mSiO_2_-DOX. (a) Single-dose Bliss analysis at 104.2 μg·mL^-1^ DOX equivalent and 300 J·cm^-2^ NIR; P < 0.01 by the Demidenko-Miller interaction analysis using n = 3 independent experiments. (b) Post-hoc Loewe isobolograms at Fa = 0.25, 0.50, and 0.75; NIR-fluence values on the ordinate represent the corresponding fixed nanoparticle-concentration/fluence pairs along the predefined photothermal treatment-intensity axis. Straight lines represent additivity, and the corresponding same-Fa combination points are shown relative to each line.

# **References**

1. M. Šoltys, P. Kovačík, M. Lhotka, P. Ulbrich, A. Zadražil, F. Štěpánek. Radiofrequency controlled release from mesoporous silica nano-carriers. Microporous Mesoporous Mater., 2016, 229: 14-21. doi: 10.1016/j.micromeso.2016.04.009

2. H. A. Hoffman, L. Chakrabarti, M. F. Dumont, A. D. Sandler, R. Fernandes. Prussian blue nanoparticles for laser-induced photothermal therapy of tumors. RSC Adv., 2014, 4: 29729-29734. doi: 10.1039/C4RA05209A

3. A. Kroll, M. H. Pillukat, D. Hahn, J. Schnekenburger. Interference of engineered nanoparticles with in vitro toxicity assays. Arch. Toxicol., 2012, 86(7): 1123-1136. doi: 10.1007/s00204-012-0837-z

4. A. L. Holder, R. Goth-Goldstein, D. Lucas, C. P. Koshland. Particle-Induced Artifacts in the MTT and LDH Viability Assays. Chem. Res. Toxicol., 2012, 25(9): 1885-1892. doi: 10.1021/tx3001708

5. T.-C. Chou. Drug Combination Studies and Their Synergy Quantification Using the Chou-Talalay Method. Cancer Res., 2010, 70: 440-446. doi: 10.1158/0008-5472.CAN-09-1947

6. E. Demidenko, T. W. Miller. Statistical determination of synergy based on Bliss definition of drugs independence. PLoS ONE, 2019, 14(11): e0224137. doi: 10.1371/journal.pone.0224137

7. A. Ianevski, A. K. Giri, T. Aittokallio. SynergyFinder 3.0: an interactive analysis and consensus interpretation of multi-drug synergies across multiple samples. Nucleic Acids Res., 2022, 50(W1): W739-W743. doi: 10.1093/nar/gkac382
